# Analysis of the Exeter V40 femoral stem prosthesis fracture: systematic review and single centre case series

**DOI:** 10.1302/2633-1462.26.BJO-2021-0025.R1

**Published:** 2021-06-28

**Authors:** Joshua W. Thompson, James Corbett, Daniel Bye, Adrian Jones, Elizabeth K. Tissingh, John Nolan

**Affiliations:** 1 Department of Trauma and Orthopaedic Surgery, Norfolk and Norwich University Hospital NHS Foundation Trust, Norwich, Norfolk, UK

**Keywords:** V40, Exeter, Failure, fracture, stem, incidence

## Abstract

**Aims:**

The Exeter V40 cemented polished tapered stem system has demonstrated excellent long-term outcomes. This paper presents a systematic review of the existing literature and reports on a large case series comparing implant fractures between the Exeter V40 series; 125 mm and conventional length stem systems.

**Methods:**

A systematic literature search was performed adhering to the Preferred Reporting Items for Systematic Reviews and Meta-Analyses (PRISMA) criteria. In parallel, we performed a retrospective single centre study of Exeter V40 femoral stem prosthetic fractures between April 2003 and June 2020.

**Results:**

There are 25 reported cases of such prosthetic fractures confined to small case series and case reports within the literature. We report an additional 19 cases to the literature (mean age 66.3 years (SD 11.7); 12 (63%) females; BMI 32.9 kg/m^2^ (SD 5.9)). The mean time from index procedure to fracture was 7.8 years (SD 3.6; 2.5 to 16.3). Exeter V40 stem fracture incidence was 0.15% and 1.21% for primary and revision arthroplasty, respectively. Incidence was significantly higher in revision arthroplasty (p < 0.001) and 125 mm length stems compared to ≥ 150 mm length stems (1.25% vs 0.13%, respectively; p < 0.001). When comparing different stem length cohorts, 125 mm short-stems were associated with stem-body fractures (92% vs 29%; p = 0.0095), earlier time to fracture (6.2 years vs 11.0 years; p = 0.0018), younger patient age at time of fracture (62.7 years vs 72.6 years; p = 0.037), and female sex (75% vs 43%; p = 0.326).

**Conclusion:**

This complication remains rare, although we report a significantly higher incidence at up to 17 years follow-up than in the literature. Short 125 mm length Exeter V40 stems undoubtedly have a role in restoring anatomy and biomechanics in smaller femoral geometries, although the surgeon has to appreciate the higher risk of stem fracture and the associated predisposing factors which may necessitate particular attention to surgical technique and planning.

Cite this article: *Bone Jt Open* 2021;2(6):443–456.

## Introduction

Since the modification of the Exeter stem system in 2000, the Exeter V40 stem (Stryker Orthopaedics, USA) has become the most common cemented femoral component used in total hip arthroplasty (THA) in the UK^1^ and worldwide.^2^ Accounting for 60% of all cemented femoral stems, with over 35,000 stems implanted in 2018 in the UK alone, the Exeter V40 stem has remained a market leader for nearly two decades.^1^

In the modern era, femoral prosthetic fractures are a recognized but rare complication of THAs.^3^ In the original design established by Ling et al^4^ over 30 years ago, the femoral implant fracture rate was as high as 3.46% and 3.92% for neck and stem prosthetic fractures, respectively. The evolution of the modern Exeter V40 stem geometry since 2000 has proved to be a reliable femoral component.^5,6^

Prosthetic fractures are rare complications, therefore reporting is limited to individual case reports and small case series. National Joint Registries and manufacturers reports have their own limitations.^7^ Causes for failure of prosthesis are multifactorial, falling into three categories: patient, surgical, and implant factors.^8^ To predict the modality and probability for implant failure, it is fundamental to evaluate all three factors. Detailed case review of implant failures are essential for the modification or development of successful implant longevity.

Our systematic review and observational study aim was to assess whether the shorter (125 mm) Exeter V40 stem systems in primary and revision THA has a higher risk of implant fracture in comparison to the conventional Exeter V40 stem series. Secondary aims were to identify predisposing factors and more accurately define incidence of implant fracture.

## Methods

### Systematic review

**Fig. 1:**
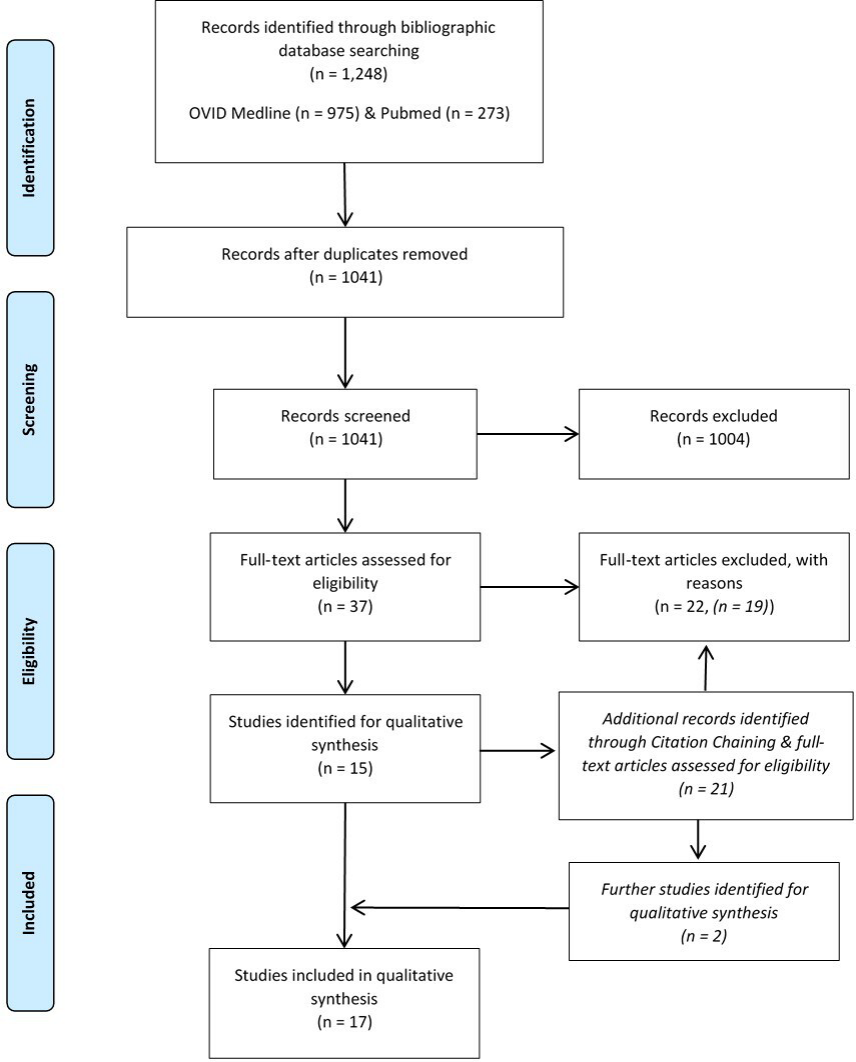
Flowchart of search strategy.^9^

A systematic literature search was performed adhering to the Preferred Reporting Items for Systematic Reviews and Meta-Analyses (PRISMA) criteria.^9^ The search criteria were defined as “Exeter” or “V40” and “fracture”. Inclusion and exclusion criteria were applied as defined in Table I. Initially, two authors (JT, JC) independently screened all search studies for relevance and eligibility; any inconsistencies or disagreements were resolved by discussion and consensus. After completion of this process, 17 articles were selected (Figure 1).

**Table I.** Inclusion and exclusion criteria.

Inclusion criteria	Exclusion criteria
Exeter V40 stem system prosthetic fractures. Inclusive of the entirety of the stem; neck (taper included) and stem. Primary or revision hip arthroplasty. There were no exclusions based on language of publication.	Non-Exeter stem fractures. Exeter Universal stem fractures. Exeter unspecified stem fractures implanted prior to 2002. No reported stem fractures. Exeter V40 periprosthetic fractures. Alternate component fractures (prosthetic head, liner, or acetabular component).*

* Exclusions were conducted by JT and JC, and any non-conformity was discussed and resolved.

## Observational study data collection

Norfolk & Norwich University Hopsital (NNUH) NHS Foundation Trust is a large tertiary hip revision centre. Searches were completed and clinical data extracted for all THA operations performed at NNUH between April 2003 and June 2020 from our centre’s electronic operational database record, Bluespier Patient Manager (Bluespier international, UK), using search criteria: “ICD 10 T84.0; mechanical complication of internal joint prosthesis” and "Operating Room Scheduling and Office System (ORSOS)” (v.3.5.2; McKesson, USA) for implant coding. The same inclusion and exclusion criteria were applied (Table I). Identified stem failures were cross referenced with National Joint Registry (NJR) data coding for “implant fracture stem” over the same period for our institution, and no additional cases were identified. Total volume of Exeter V40 stems implanted during the study period was extracted from our electronic operative database. As per trust policy, THA patients were not routinely followed-up beyond two years, thus incidence data calculated from this study represents a minimum fracture rate.

## Radiological assessment

Anteroposterior and lateral radiographs were evaluated on a consensus basis by two authors not involved with the index procedure. Pre-fracture, post-fracture, and post-revision radiographs were evaluated for cementation quality according to Barrack’s classification^10^ and Gruen Zones^11^ of radiolucency. Stem positioning was measured relative to the medullary canal axis.

### Demographic data

We collected the following patient demographics: age, sex, BMI, American Society of Anesthesiologists (ASA) grade, and preoperative diagnosis.

### Statistical analysis

The study was given institutional approval (study reference 8-10-003) and conducted according to STROBE guidelines for observational studies.^12^ Statistical analysis was undertaken using RStudio 1.3.959 (R Foundation for statistical Computing, Austria). Continuous data were tested for distribution, with normal distributed data presented as mean, standard deviation, and range, and differences between groups were tested using the unpaired *t*-test. Fisher’s exact test was used for categorical data as appropriate.

## Results

Excluding NJR reports, there have been 25 reported cases of such prosthetic fractures (Table II), 18 primary THAs, and seven revision THAs (mean age 68.2 years (standard deviation (SD) 12.4; 40 to 87); 13 (53%) females; BMI 35.1 kg/m^2^ (SD 7.0; 25 to 47)).^2,5-7,13-25^ The mean time from index procedure to fracture was 6.3 years (SD 2.4; 2 to 10). Indication for index procedure and location of the prosthetic femoral fracture is shown in Table III.

**Table II.** Results of the Exeter V40 stem fractures systematic review.

Article, yr	Study design	Fracture location	Stem	Head + offset	1° or revision	Time to #, yrs	Age, yrs	Sex	BMI, m^2^/kg	Revision technique	Reconstruction stem	Recon head	Findings/comments
O'Neill et al, 2011^13^	CR	Neck (base)	44/00/125	N/S	R	5	71	F	27.8	C-in-C	44/00/125	N/S	“Slight varus alignment, but there was good medial support and good cement mantle proximally”. The fracture originated and propagated from the introducer hole.
Davies et al, 2013^14^	CS	Stem (mid) Stem (mid) Stem (mid) Stem (mid)	35.5/125 35.5/125 44/0/150 44/1/150	26 + 0 N/S + 4 28 + 0 N/S	P P P R	5 2 4 10	69 64 57 48	F M M M	44 47 34 33	ETO C-in-C Non-op ETO	Restoration PS (Stryker) CPT (Zimmer) size 0 Palliate (liver cancer) Restoration PS (Stryker)	26 + 4 3.5 N/A 32 + 4	Stryker analysis: fracture due to metal fatigue (case 1) “All primaries considered to have low neck cuts & the revision had a fracture through the calcar & significant greater trochanter bone loss.” “All patients were obese, with small stems but not considered undersized”.
Hamlin et al, 2014^15^	CR	Neck (prox)	44/2/150	N/S + 0	R	5	76	M	45	C-in-C	44/1/150	N/S	Stryker analysis: fatigue failure initiated at the lateral side of the trunnion-neck junction. “Morbid obesity identified as an obvious modifiable contributary factor
Chung et al, 2015 ^16^	CR	Stem (mid)	33/115	28 + 5	P	6	78	F	31.6	ETO	Long solution stem (Depuy)	N/S	“Fracture of the proximal medial cement mantle”. “Neck-cup impingement identified and an anterior notch distal to fracture line”.
McSorley et al, 2015^17^	CR	Stem (prox)	37.5/0/150	28 + 4	P	5.5	40	F	> 30	Cortical window	37.5/0/150	28 + 4	“Loose proximal cement mantle with a well-fixed stem distally.” “Relatively undersized femoral stem. Young and obese patient. Index procedure for neck of femur fracture.”
Facek et al, 2016^18^	CR	Neck (base)	44/0/150	28 + 0	P	6	70	F	< 25	C-in-C	44/00/125	N/S	SEM analysis: fracture initiated from anterosuperior edge close to the insertion guide ‘dimple’. Stem surface revealed ‘dimple rupture’, cracking, beach marks and areas of fatigue striations, common to fatigue failure. “Augmented by champagne glass femur morphology and poor proximal support.”
Reito et al, 2016^19^	CS	Neck (prox) Neck (prox) Neck (prox)	44/3/150 44/2/150 44/1/150	36 + 5 40 + 8 36 + 10	P P R^2^	5.3 4 2.8	63 87 76	F M M	31 27 38	C-in-C C-in-C Cemented revision	44/00/125 44/00/125 50/1/150	36 + 5 40 + 8 36 + 5	Posterior notch distal to the fracture line on the stem (Case 1). Anterior notch distal to the fracture line on the stem (Case 3). “All cases had a well fixed stem.” “Concluded increased leverage caused by additional offset in larger head sizes may be associated increased risk of stem fracture (0.3%).” Incidence: 3/2,521 (0.12%) 2002 to 2014.
Sanchez et al, 2016^20^	CR	Stem (mid/dist)	37.5/150	48 (bipolar)	P	8	82	F	25	Uncemented revision	Diaphyseal anchor stem (Solution)	N/S	“Bipolar hemiarthroplasty performed for subcapital neck of femur fracture. Proximal debonding of cement mantle due to poor cementation technique likely cause of fatigue fracture.”
Garala et al, 2018 ^2^	CS	Junction Neck (prox) Stem (mid)	44/3/150 44/1/150 37.5/125	28 + 0 28 + 4 28 + 4	P P P	10 7 4	63 61 64	M F F	37.3 95 kg 107 kg	C-in-C C-in-C C-in-C	50/1/125 44/00/125 44/00/125	28 + 0 28 + 4 28 + 0	“Fracture through the insertion guide hole” (case 1). “Fracture distal to trunnion, in an obese patient and plus sized head which would have led to a greater moment arm of the implant” (case 2). “Well-fixed distal stem and may have been undersized” (case 3).
Westerman et al, 2018^5^	RS	Neck	44/3/150	N/S	P	9.5	N/S	N/S	N/S	N/S	N/S	N/S	“Impingement of the femoral neck against the titanium backing of the ceramic acetabular component noted.” Incidence; 1/540 (0.19%)
Shaw et al, 2018^21^	CR	Stem (mid)	37.5/150	N/S	P	9	71	N/S	N/S	ETO	Restoration (Stryker)	28 mm	Stryker analysis; fatigue failure with the origin on the anterolateral surface. Bending stresses secondary to proximal loosening and firm distal fixation.
Woodbridge et al, 2019^22^	RS	Stem (mid)	44/00/ 125	N/S	R	N/S	N/S	N/S	N/S	N/S	N/S	N/S	“Septic lysis with 7 cm calcar defect & unsupported proximal segment. Patient refused revision for infection prior to fracture.”
Moloney et al, 2019^23^	CR	Neck (base)	44/2/150	36 + 0	P	6	87	M	30.1	C-in-C	C-stem size 1 (Depuy)	36 + 5	“Fracture propagated from the introducer site” *London retrieval analysis*: stress riser at the insertion site, cyclical loading and unloading of stem leading to growth of the crack and revision of the implant prior to full separation at the fracture site.
Berg et al, 2020^24^	RS	Neck (base)	44/00/ 125	N/S	R	5.6	N/S	N/S	40	N/S	44,/00/125	N/S	“High BMI with previous revision surgery for neck fracture of the primary THA C-stem femoral component“.
Matar et al, 2020^25^	RS	Stem (mid) Stem (mid) Stem (prox) Stem (prox)	N/S N/S N/S N/S	N/S N/S N/S N/S	R P P P	9 ~8.6 ~8.6 ~8.6	N/S N/S N/S N/S	… … … …	N/S N/S N/S N/S	Cortical window Cortical window ETO ETO	Uncemented revision Primary cemented stem Uncemented revision Uncemented revision	N/S N/S N/S N/S	Revision THA; “No remaining trochanter at index procedure”. Primary THAs; “Proximal debonding of cement mantle likely caused by cantilever bending stresses in a distally well-fixed stem”.
**National Joint Registry Studies**													
	**Data reviewed**	**Number of stem fractures**	**Primary or revision**	**Total implants**	**Fracture incidence, %**			**Stem type**		**Observations/conclusion**		**Study design**	
Kazi et al, 2019^6^	UK NJR 2003 to 13	30	Primary	176,189	0.017			V40 stems		All composite beam cemented stems 0.008%	Alternate taper-slip 0.023%		NJR comparison study of taper slip and composite beam femoral stems. The Exeter taper slip stem demonstrated significant survival advantage versus all other stems.
Rickman et al, 2020 ^7^	AUS NJR 1999 to 2015	41	Primary	76,120	0.052			Universal (0.4%) & V40 (99.6%) stems		“Overall incidence is very low.”	Increased risk associated with < 70 yrs, male and exchangeable necks.		AOANJRR analysis for risk factors for femoral stem breakage.

#, fracture; AOANJRR, Australian Orthopaedic Association National Joint Replacement Registry; C-in-C, Cement-in-cement; CR, case report; CS, case series; dist, distal; ETO, extended trochanteric osteotomy; F, female; M, male; mid, middle; N/A, not applicable; NJR, National Joint Registry; NOF, neck of femur; N/S, not specified; N/S, not significant; P, primary; prox, proximal; R, revision; R*^2^*, second revision; RS, retrospective study; SEM, scanning electron microscopy; stem 44/100/125, 44 mm offset/size 00/125 mm length.; THA, total hip arthroplasty

**Table III.** Norfolk & Norwich University Hopsital (NNUH) and systematic review of Exeter V40 stem fractures, summary of indications for index procedure and femoral prosthetic fracture location.

Indication for index surgery	NNUH case series, n (%)	Literature systematic review, n (%)
**Primary arthroplasty, n**	11	18
Osteoarthritis	10 (53)	12 (48)
Inflammatory arthritis	1 (5)	0 (0)
Neck of femur fracture	0 (0)	2 (8)
Unspecified	N/A	4 (16)
**Revision arthroplasty, n**	**8**	**7**
Aseptic loosening	0 (0)	3 (12)
Instability	0 (0)	1 (4)
Metal-on-metal	6 (32)	0 (0)
Conversion from hemiarthroplasty	2 (11)	0 (0)
Conversion from internal fixation	0 (0)	1 (4)
prosthetic fracture	0 (0)	1 (4)
prosthetic joint infection	0 (0)	1 (4)
**Femoral prosthetic fracture location**		
Neck	6 (32)	11* (44)
Trunnion junction	3 (16)	5 (20)
Neck base	3 (16)	5 (20)
Stem	13 (68)	14 (56)
Proximal third	1 (5)	3 (12)
Middle third	10 (53)	10 (40)
Middle-to-distal third junction	2 (11)	1 (4)
Distal third	0 (0)	0 (0)

* One unspecified location of a neck fracture.^5^

N/A, not applicable.

Stem extraction and femoral revision techniques are summarized in Table IV. Five studies reported metallurgical analysis completed by Stryker^14,15,21^ or independent retrieval analysis centres,^18,23^ with no material or dimensional defects present with the Exeter V40 stem range. One reported case of stem fracture was associated with infection.^22^

**Table IV.** Norfolk & Norwich University Hopsital (NNUH) and systematic review Exeter V40 stem fractures, summary of femoral stem extraction and revision techniques.

Variable	NNUH case series, n (%)	Literature systematic review, n (%)
**Femoral stem extraction technique**		
Proximal drilling	14 (74)	12 (48)
Extended trochanteric osteotomy	2 (11)	6 (24)
Implant exchange	2 (11)	0 (0)
Cortical windowing	0 (0)	3 (12)
Nonoperative	1 (5)	1 (4)
Unspecified	N/A	3 (12)
**Femoral revision technique**		
Cemented	9 (47)	3 (12)
Femoral impaction grafting + cemented	1 (5)	0 (0)
Cement-in-cement	4 (21)	10 (40)
Un-cemented	2 (11)	8 (36)
Implant exchange	2 (11)	0 (0)
Nonoperative	1 (5)*	1 (4)
Unspecified	N/A	3 (12)
**Acetabular component**		
Component revised	3 (16)	N/A
Liner exchange	7 (37)	N/A

* Patient declined operation due to concerns surrounding COVID-19 pandemic and associated perioperative risks.

N/A, not applicable.

NJR studies reported incidence of Exeter V40 stem fracture following primary THA between 0.017% (30/176,189) and 0.052% (41/76,120) at mean follow-up 4.2 years (0 to 12) and 5.3 years (0 to 13), respectively.^6,7^ Neither study provided analysis of demographic data, operative data, site, or mechanism of fracture.

## Observational study

This single-centred, retrospective case series included 19 patients (19 hips) (mean age 66.3 years (SD 11.7; 43 to 85); 12 (63%) females; BMI kg/m^2^ 32.9 (SD 5.9; 24 to 44)) with fractured primary (n = 11) or revision (n = 8) femoral Exeter V40 stems in our unit and managed by six revision arthroplasty surgeons between 2003 and 2020 (Table V). In all, 19 patients were identified through local electronic databases. All patients presented between April 2012 and July 2020; the mean time from index procedure to fracture was 7.8 years (SD 3.6; 2.5 to 16.3).

**Table V.** Results of the Exeter V40 stem fractures at Norfolk & Norwich University Hopsital from 2003 to June 2020.

Case no.	Sex	Age, yrs	BMI, kg/m^2^	Year of stem fracture revision	Year of previous surgery	Year of primary surgery	Time to #, yrs	Indication for index surgery	Fracture site	Stem	Femoral head size + offset, mm	Stem extraction technique	Femoral revision technique	Femoral revision component revised to
**Primary total hip arthroplasty**														
1	M	67	41.4	2015	2010	P	5.8	OA	Midstem	35.5/125	32 + 0	ETO	Cemented	44/0/150
2	M	77	38	2016	2006	P	9.9	OA	Neck (trunnion)	44/3/150	28 + 4	Proximal drilling	Cemented	50/2/150
3	F	82	36.5	2016	2003	P	13.2	OA	Neck (base)	44/2/150	28 + 0	Proximal drilling	Cement-in-cement	44/00/125
4	M	73	31.9	2017	2005	P	11.3	OA	Neck (base)	44/2/150	32 + 0	Implant exchange	Implant exchange	44/2/150
5	M	43	25	2018	2011	P	6.9	IA	Midstem	35.5/125	32 + 0	Proximal drilling	Femoral impaction grafting + cemented	44/0/150
6	F	63	30	2018	2009	P	8.8	OA	Midstem	35.5/125	32 + 4	Proximal drilling	Cemented	44/1/150
7	F	57	30.5	2019	2009	P	10.0	OA	Proximal stem	35.5/125	32 + 0	Proximal drilling	Cemented	44/0/150
8	F	48	26.5	2019	2014	P	5.3	OA	Midstem	35.5/125	28 + 0	Proximal drilling	Cement-in-cement	44/1/150
9	F	58	32.4	2019	2009	P	10.9	OA	Midstem	35.5/125	32 + 0	ETO	Un-cemented	Restoration Modular
10	F	69	36.2	2020	2017	P	2.5	OA	Middle/distal third stem	35.5/125	28 + 4	Proximal drilling	Cemented	44/0/150
11	F	62	24.4	2020	2004	P	16.3	OA	Neck (trunnion)	37.5/1/150	28 – 4	Implant exchange	Implant exchange	37.5/1/150
**Revision total hip arthroplasty**														
12	F	72	N/S	2012	2008	2001	3.9	M-o-M	Midstem	35.5/125	36 + 4	Proximal drilling	Cemented	44/2/150
13	F	68	38.3	2014	2007	2002	6.6	M-o-M	Midstem	35.5/125	32 + 4	Proximal drilling	Cement-in-cement	44/2/150
14	F	69	37	2017	2011	2011	5.8	Hemi- arthroplasty (infected)	Neck (trunnion)	44/2/205	28 + 4	Proximal drilling	Cement-in-cement	44/2/205
15	F	46	44	2017	2009	1992	7.8	Hemiarthroplasty	Midstem	35.5/125	32 + 4	Proximal drilling	Cemented	44/1/150
16	M	71	29	2018	2007	1991	11.2	M-o-M	Middle/distal third stem	44/2/150	32 + 0	Proximal drilling	Un-cemented	Restoration Modular
17	F	76	25	2018	2011	2003	6.7	M-o-M	Midstem	35.5/125	32 + 4	Proximal drilling	Cemented	44/1/150
18	M	85	32.9	2020	2015	2002	4.9	M-o-M	Neck (base)	44/00/125	32–4	Proximal drilling	Cemented	44/4/150
19	M	74	N/S	2020	2007	1999	12.9	M-o-M	Midstem	44/1/150	36 + 0			

44/3/150, 44 mm offset/size 3/150 mm length; #, fracture; ETO, extended trochanteric osteotomy; IA, inflammatory arthritis; MoM, metal-on-metal; N/S, not specified; OA, osteoarthritis; P, primary.

Patient demographics stratified by stem type are displayed in Table VI. All patients had their index operation performed by an arthroplasty consultant from our unit. Three patients had their index operation at our local private hospital, either under the NHS service or privately and were therefore excluded from incidence data calculations.

**Table VI.** Exeter V40 stem fractures (n = 19) managed at Norfolk & Norwich University Hopsital between April 2003 and July 2020 categorized according to stem characteristics.

Stem characteristic	Short 125 mm stem, n (%)	Standard 150 mm+ stem, n (%)	p-value*	Stem body fracture, n (%)	Neck fracture, n (%)	p-value*	Primary stem, n (%)	Revision stem, n (%)
Total cases	12 (63)	7 (37)		13 (68)	6 (32)		11 (58)	8 (42)
Primary stem	7 (58)	4 (57)	1.000	7 (54)	4 (67)	0.659	N/A	N/A
Revision stem	5 (42)	3 (43)		6 (46)	2 (33)		N/A	N/A
M-o-M revision stem	4 (33)	2 (29)	1.000	5 (38)	1 (17)	0.605	N/A	6 (75)
Stem fracture	11 (92)	2 (29)	0.0095	N/A	N/A		7 (64)	6 (75)
Neck fracture	1 (8)	5 (71)		N/A	N/A		4 (36)	2 (25)
Short length stem	N/A	N/A		11 (8)	1 (17)	0.0095	7 (64)	5 (63)
Standard length stem	N/A	N/A		2 (15)	5 (83)		4 (36)	3 (38)
Males	3 (25)	4 (57)	0.326	4 (31)	3 (50)	0.617	4 (36)	3 (38)
Females	9 (75)	3 (43)		9 (69)	3 (50)		7 (64)	5 (63)
Age, yrs	62.7	72.6	0.037	62.46	74.67	0.0296	63.55	70.13
BMI, kg/m^2^	32.9	32.8	0.969	32.6	33.5	0.779	32.1	34.4
ASA grade	2.2	2.5		2.3	2.4		2.4	2.3
Stem alignment, varus°	2.58	1.7	0.368	2.46	1.8	0.533	2.27	2.25
Time to stem fracture, yrs	6.2	11.0	0.0018	7.00	9.67	0.1509	8.64	6.75
Time to stem fracture, mnths	80.1	138.1		91.62	122.83		110.09	89.63

* Continuous data was tested using the unpaired *t*-test and categorical data using Fisher’s exact test as appropriate.

MoM, metal-on-metal; N/A, not applicable.

Preoperative diagnoses and indications for revision procedure associated with the index surgery are shown in Table III. There were 11 stem fractures following primary THA; the preoperative diagnoses for the index surgery included primary osteoarthritis (n = 10; 91%) and inflammatory arthritis (n = 1; 9%). Eight patients sustained fractures of revision stems; indications for revision prior to stem fracture included primary metal-on-metal THA (n = 6; 75%) and conversion from hemiarthroplasty to THA (n = 2; 25%). All revisions for metal-on-metal THA were single stage revisions using a cement-in-cement technique and five with evidence of adverse reaction to metal debris (ARMD) at time of revision. Notably, only one case involving a 44/00/125 neck fracture was associated with preceding elevated serum chromium and cobalt levels. Of the two hemiarthroplasty conversion to THA patients, one patient had a two-stage revision for early infected hip hemiarthroplasty implanted in Egypt, following a traumatic subcapital neck of femur fracture on holiday, and the other patient had a single stage revision 25 years following a Bateman bipolar hemiarthroplasty in her twenties secondary to slipped capital femoral epiphysis.

There was no macroscopic, histological, or biochemical evidence of infection at time of revision for stem fracture or at follow-up for all cases.

### Prosthetic fracture configuration

Location of the prosthetic femoral fracture is shown in Table III. Mean stem alignment was + 2.26° varus. Inadequate proximal support was evident in 62% (n = 8) of stem-body fractures and 33% (n = 2) of neck fractures at the most recent radiographs preceding fracture. A summary of postoperative radiological assessment is shown in Table VII.

**Table VII.** Summary of radiological assessment prior to stem fracture at Norfolk & Norwich University Hopsital.

Barrack’s grade	A	B	C		D		
No. of hips	3	10	6		0		
**Paprosky score**	**0**	**I**	**II**		**IIIA**	**IIIB**	
No. of hips	7	7	4		1	0	
**Stem axis**	**Valgus** X < -2°	**Neutral** -2°< X < + 2°	**Mild varus** + 2° < X ≤ + 3°		**Moderate varus** + 3°< X ≤ + 5°	**Severe varus** X > + 5°	
No. of hips	0	9	7		3	0	
**Cement mantle lucency Gruen zones**	**Zone 1**	**Zone 2**	**Zone3**	**Zone 4**	**Zone 5**	**Zone 6**	**Zone 7**
No. of hips	5	0	1	1	1	1	7
**Cement mantle lucency Gruen zones**	**Zone 8**	**Zone 9**	**Zone 10**	**Zone 11**	**Zone 12**	**Zone 13**	**Zone 14**
No. of hips	3	1	0	1	1	2	1

### Implant type

**Fig. 2:**
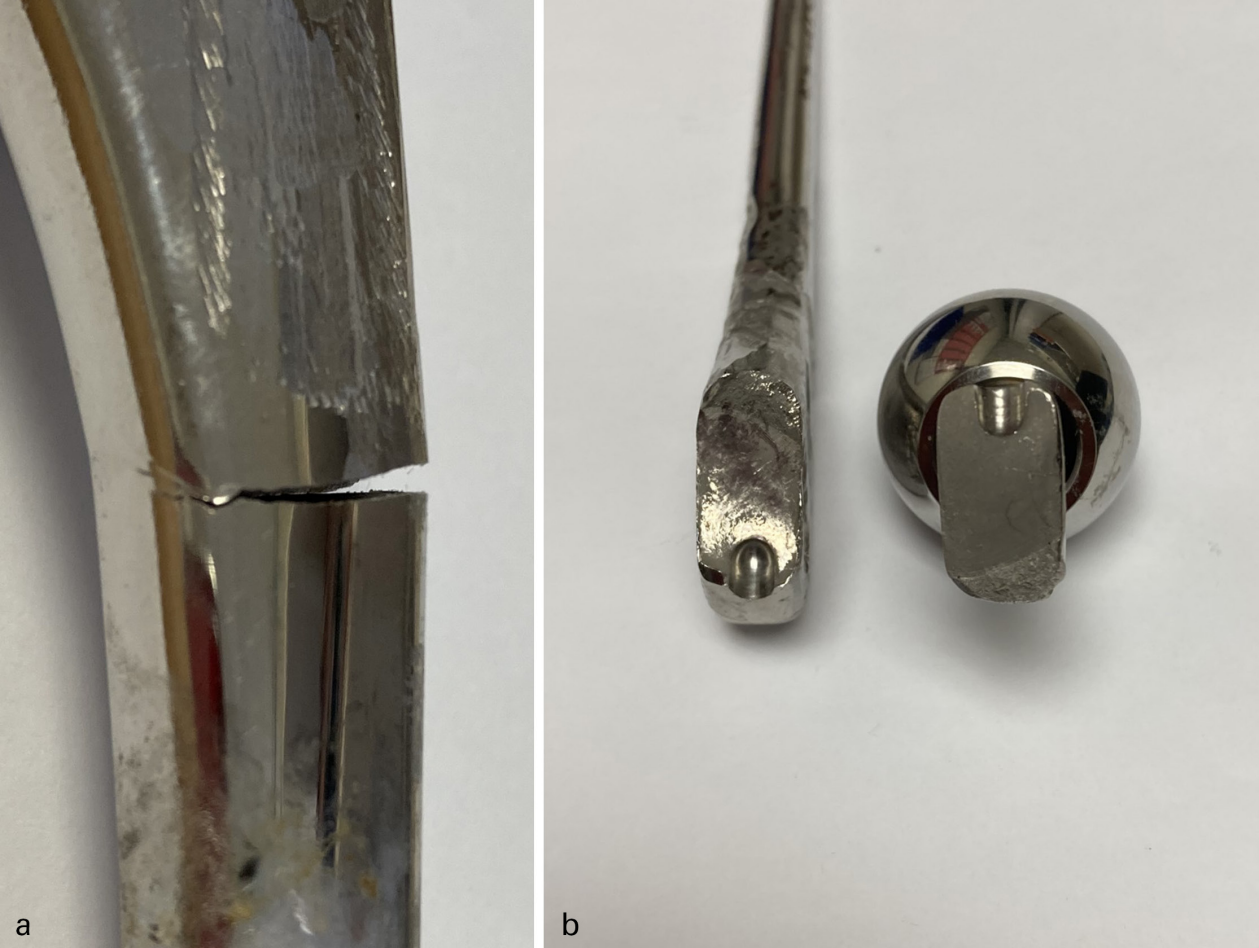
Norfolk & Norwich University Hopsital macroscopic photographs of a fractured a) Exeter 35.5/125 V40 stem at the proximal-midstem (incomplete fracture) ten years post implantation (corresponds to Figure 3 radiographs). Scratches to the proximal stem were caused by the cebetome during difficult explant. b) Exeter 44/0/150 V40 stem at the neck base, propagating from the insertion dimple, 13 years post-implantation (the stem was implanted at a local district general hospital and thus not included in study data).

**Fig. 3:**
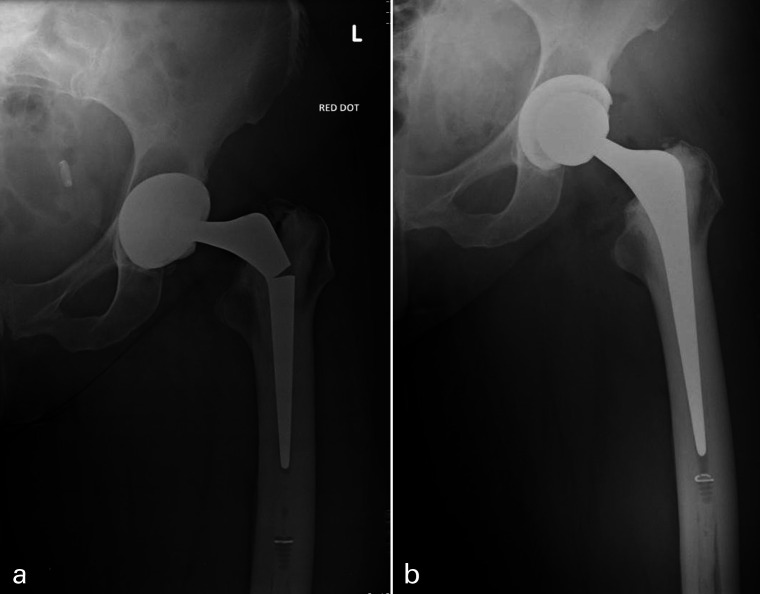
a) Anteroposterior radiographs of left hip with an example of a proximal third stem-body fracture of a cemented Exeter V40 35.5 mm offset stem at ten years post implantation, demonstrating moderate varus malpositioning and incomplete stem fracture. b) Postoperative radiographs following cemented revision using a larger 44/0/150 V40 stem.

**Fig. 4:**
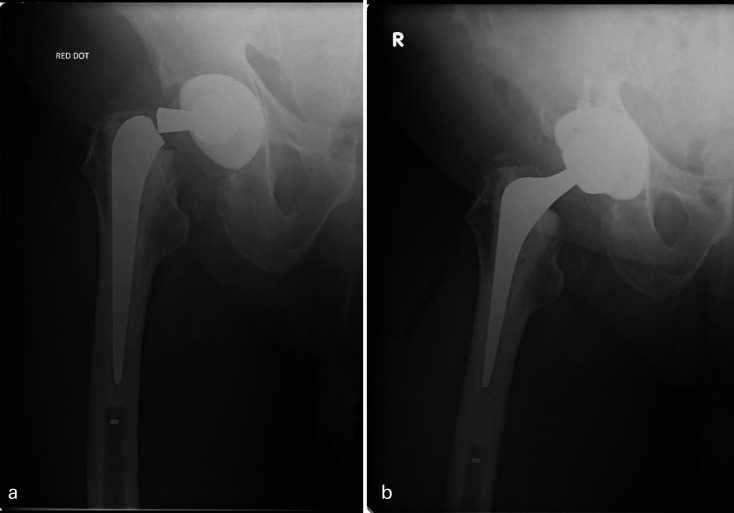
a) Anteroposterior radiographs of right hip with an example of a base of neck fracture of a cemented Exeter 44/2/150 V40 stem at 13 years post-implantation, moderate radiolucency in Gruen zone 7 evident. b) Postoperative radiographs following cement-in-cement revision using the short 44/00/125 V40 revision stem.

**Fig. 5:**
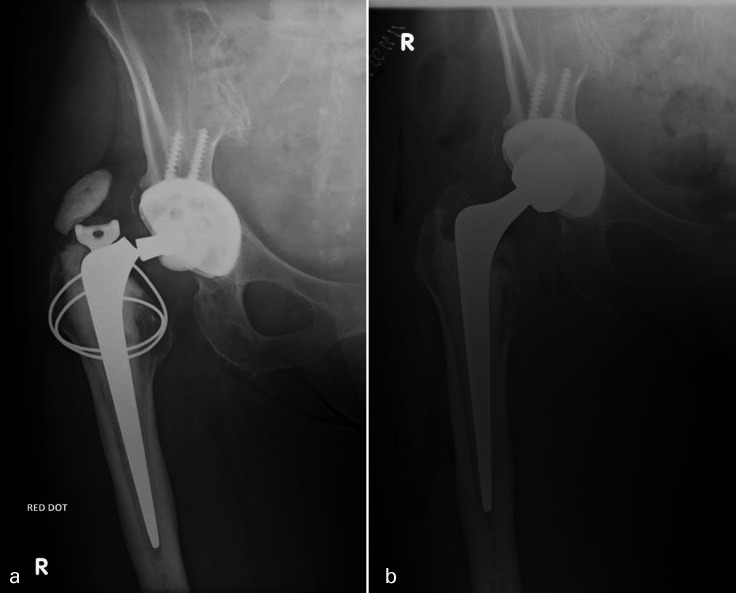
Anteroposterior radiographs of right hip with an example of a neck fracture of a primary cemented Exeter V40 37.5/150 at 16 years post implantation. a) Hip radiograph at 16 years with neck fracture demonstrating a distally well-fixed stem. b) Postoperative radiographs following revision by implant exchange into a well bonded and intact cement mantle using same size 37.5/150 V40 stem.

Individual patients’ implant types are specified in Table V. In all, 12 prosthetic fractures were 125 mm short-stem prostheses (Figure 2 and Figure 3) and seven fractures of standard 150 mm stem or longer (Figure 2, 4 and Figure 5). In all, 8/19 (42%) implant failures used a head of an increase offset (+4 mm) of which two were neck fractures (both 150 mm stems) and six were stem body fractures (one 205 mm stem and five 125 mm short stems). When comparing stem length, 125 mm short-stem fractures were associated with stem-body fractures (92% vs 29%; p = 0.0095), shorter time to fracture (6.2 vs 11.0 years; p = 0.0018), younger patient age at fracture (62.7 vs 72.6 years, p = 0.0373), and female sex (75% vs 43%; p = 0.326) (Table IV).

### Incidence

During the study period, 5,370 primary and 662 revision THAs involving the Exeter V40 stems were implanted at NNUH (Table VIII). The incidence of Exeter V40 stem fracture was 0.15% and 1.21% for primary and revision arthroplasty, respectively (Table VIII). The incidence of stem fracture was significantly greater in 125 mm short-stem prosthesis (1.25% vs 0.13%; p < 0.001, Fisher's exact test) and revision arthroplasty (1.21% vs 0.15%; p < 0.001, Fisher's exact test). Specifically, for both 125 mm short-stem prosthesis and standard 150 mm stem or longer the incidence of stem fracture was significantly higher for revision arthroplasty versus primary (4.27% vs 0.66%; p = 0.009, Fisher's exact test, and 0.55% vs 0.08%; p = 0.028, Fisher's exact test, respectively). The highest incidence of stem fracture was recorded in revision THAs involving the 35.5/125 mm stems (8%) and 44/00/125 mm stems (1.64%).

**Table VIII.** Exeter V40 stems implanted at Norfolk & Norwich University Hopsital between April 2003 and January 2018, and incidence of stem fracture.

Stem length, mm	Total primary stems implanted, n	Stem fractures, n	Incidence, %	Total revision stems implanted, n	Stem fractures, n	Incidence, %	p-value*	Total stems implanted, n	Stem fracture, n	Incidence, %	p-value*
115	1	0	0.0	1	0	0		2	0		
**125**	**605**	**4**	**0.661**	**117**	**5**	**4.274**	** 0.009**	**722**	**9**	**1.247**	** < 0.001**
35.5/125	589	4	0.679	50	4	8.000		639	8		
37.5/125	1	0	0.0	3	0	0.0		4	0		
37.5/1/125	1	0		3	0	0.0		4	0		
44/00/125	12	0	0.0	61	1	1.639		73	1		
44/1/125	1	0		0	0			1	0		
50/1/125	1	0		0	0			1	0		
**150+**	**4,764**	**4**	**0.084**	**544**		**0.551**	** 0.028**		**7**	**0.132**	** < 0.001**
150	4,730	4	0.085	319	2	0.627		5,049	6		
205+	34	0	0.0	225	1	0.444		265	1		
**Total**	**5,370**	**8**	**0.149**	**662**	**8**	**1.209**	**≤ 0.001**	**6032**	**16**		

44/00/150 = 44 mm offset/size 00/125 mm length.Incidence of stem fracture was calculated for all Exeter V40 stems implanted from April 2003 to January 2018, with a minimum time from implantation of 30 months.

* Fisher's exact test.

### Revision technique

Stem extraction and femoral revision techniques are summarized in Table IV. All 12 revision surgeries for the fractured short 125 mm stems were revised with a longer and larger stem (Table V and Figure 3). Three patients (50%) with fractured standard-length stems were revised using the same size Exeter V40 stem as index procedure (Figure 5). With regard to the remaining three cases, one revision surgery was revised to a larger Stryker Restoration Modular stem, one revised with an Exeter V40 44/00/125 short revision stem (Figure 4), and the final case revised to a larger offset 50/2/150 stem from a 44/3/150 stem.

## Discussion

Femoral prosthetic stem fractures remain a rare complication following THA. The incidence in primary (0.15%) and revision arthroplasty (1.21%) in our case series is higher than in the reported literature (0.017% to 0.052% and 0.99%, respectively).^2,6,7,26^ Few reported cases of Exeter V40 stem fractures (25 patients) are available within the literature for analysis. Our study reports an additional 19 cases to further help understand the mechanism of fractures, identify associated and potentially preventable risk factors, as well as better establish the incidence of stem fracture within this femoral implant design.

Initial concerns were raised with the original Exeter Universal shorter stems having higher risk of mechanical overload, fatigue failure, premature stem breakage, or dramatic clinical failures.^27,28^ More recently, multiple studies in Asia and the UK demonstrated survival rates of 100% for aseptic loosening for the Exeter Universal 35.5 mm shorter stems at ten and eight years, respectively.^27,29^ The success of the V40 Exeter short revision stem (44/00/125) introduced in 2005 is well published in providing an implant which facilitates restoration of leg length, offset and stability, while limiting bone loss secondary to excess reaming in revision^22,24,30^ and recently primary THA.^30^

Large NJR studies comparing short and standard Exeter stem survivorship (Exeter V40 and Universal stem systems) for primary THA medium-term outcomes report contrasting outcomes for all cause revision rate.^31,32^ Choy et al’s^31^ Australian NJR (1999 to 2010)-based study demonstrated no significant difference (3.4% vs 3.5% at seven years from implantation). Conversely, Wyatt et al’s^32^ review of the New Zealand NJR (1998 to 2018) concluded there was a significantly higher all-cause revision rate for short-stems (0.92/100 vs 0.55/100 components-per year) and specifically for aseptic loosening cohort (19.6% vs 7.2%). Interestingly, Wyatt et al^32^ reported no femoral implant fractures (41,854 THAs), whereas Choy et al^31^ reported ten standard stem (0.03%) and one short stem (0.07%) fractures (36,266 THAs).

The shorter Exeter stems were designed to address femora geometries that are tighter, fluted, or smaller, and subsequently have higher prevalence in treatment of dysplastic hips.^32^ It is likely underlying diagnosis also impacts implant survivorship. This may have impacted two (17%) of our short-stem fractures, primary arthroplasty performed for inflammatory arthritis and developmental dysplasia of the hip. Bolland et al^8^ reported prosthesis fractures were four-fold as common within the 35.5 mm offset stems (3.94/10,000) and smaller stems (≤ 44/1/150) accounted for 84% of primary stem fractures of the Exeter Universal series.

## Mechanism of fracture

Femoral stem fracture retrieval analysis studies of the V40 predecessor, the Exeter Universal stem, have hypothesized that plastic deformation of the prosthesis through cantilever bending or torsion and mechanical overload, are biomechanically responsible for implant failure at the femoral stem and neck, respectively.^8^

In our cohort, there was no history of significant trauma preceding stem fractures. Stem fractures at the neck occur via mechanical overload or fatigue failure in a well-fixed stem with a bonded and intact cement mantle.^2,8,17^ Within the V40 range, multiple predisposing factors have been proposed, associated with both surgical factors (notch sensitivity, increased offset and femoral head size, propagation from the insertion hole,^2,5,13,18,19^) and patient factors (higher activity level and raised BMI).^15,19,24^ In our study, neck fractures were associated with standard-length stems (83%) and raised BMI (83%). Previous metallurgical studies of Exeter stem neck fractures demonstrate ductile deformation highly indicative of a high-cycle low-stress fatigue failure.^8^ In our cohort, mean time to neck fracture was 9.67 years (SD 4.3; 4 to 16) in keeping with this mechanism. In contrast to other studies, only 33% (n = 2) of neck fractures were associated with increased offset.^8,19^

Fractures of the stem body have been traditionally attributed to cantilever bending and fatigue failure. Referring back to the hypothesis by Wroblewski,^33^ “fracture of the stem is but a dramatic presentation of the end result of loosening of the proximal part of the stem in the presence of distal fixation”. In all, 93% of previously described V40 stem-body fractures at the time of fracture report inadequate proximal support, compared with 62% of stem-body fractures and 33% of neck fractures managed in our unit.^14,16-18,20-22,25^

There was no intraoperative or radiological evidence at the time of the initial revision procedures to suggest inadequate proximal femoral support. Nonetheless, we do hypothesize that the revision arthroplasty cases are susceptible to accelerated cantilever bending resulting from a proximal femoral support more prone to degrade with time due to possible ARMD or osteolysis. This hypothesis is supported by the 8% incidence of stem failure seen with the smaller V40 35.5/125 mm in revision cases, which are less able to tolerate prolonged cantilever forces. Whether this stem body failure is more likely in cement-in-cement revision versus re-cementing techniques or bone impaction grafting techniques is unclear from this study, though smaller V40 stems would be more commonly utilized in cement-in-cement revisions.

**Fig. 6:**
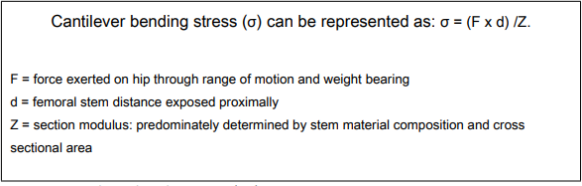
Cantilever bending stress.^17^

In relation to Figure 6, cantilever stress linearly increases with distance of femoral stem exposed proximally, highlighting the importance of good proximal cementation and bone stock. Biomechanically it is established that femoral stem diameter exponentially influences the section modulus to the third power and ultimately threshold to prosthetic fracture.^34^

Femoral canal morphology dictates stem size and cement mantle thickness, both contributory factors to risk of stem fracture.^35^ Undersizing can lead to stem fractures via three separate mechanisms: increased micromotion, higher risk of varus malpositioning, and lower threshold to fatigue failure.^14,17^ Although identified as a predisposing risk factor throughout the literature search of V40 stem fractures, no previous reported cases contributed undersizing as a cause specific to their reported fractures. In our study, all 12 short-stems were revised to larger stems. 33% of previous reported V40 fractures occurred in short-stems compared to 63% in our cohort.

Varus positioning of the femoral component is a well-established risk factor for aseptic loosening, proximal bone osteolysis, periprosthetic, and prosthetic fracture. Overall, 25% (n = 8) and 7% (n = 2) of reported stem-body and neck fractures of the Universal Exeter stem, respectively, were associated with stem varus malpositioning.^8^ In our study, mild varus (≥ 2 to 3°) and moderate varus (> 3 to ≤ 5°) positioning was present in 37% (n = 7) and 16% (n = 3) of prosthetic fractures, respectively. Varus stem positioning was most abundant in the short-stem (67% vs 33%) and stem-body fracture (62% vs 33%) cohorts. Neither were statistically significant.

Harrington et al^35^ identified BMI had the largest effect on peak strain of the proximal cement mantle and consequently advised cemented femoral stems should be avoided in high BMI patients with small medullary canal due to high cement stresses. Mean BMI in our study was significantly greater than the NJR (32.9 kg/m^2^ vs 28.8 kg/m^2^, respectively).^1^ Femoral geometries are not changing and thus with expected rises in BMI we will not only see significant financial healthcare implications but also a likely increase in the incidence of femoral prosthetic fracture.

## Fracture incidence

The incidence of surgical complications are commonly reported at variable rates.^3,6-8,19^ Sadoghi et al^3^ reported lifetime risk of stem fracture from clinical studies was 6.4-fold that of values from registries (0.77% vs 0.12%, respectively).^3^ Registry data publishing specific Exeter V40 stem fracture in primary arthroplasty incidence ranges from 0.017% to 0.052%;^6,7^ however, more recent registry data reports significantly higher fracture in the 44/00/125 (0.99%) short revision stem.^26^ We also report a significantly higher rate of stem fracture incidence within a large tertiary unit; total incidence of 0.15% and 1.21% for primary and revision arthroplasty, respectively. This is at least three-fold higher than previous NJR data reports on the Exeter V40 stem and at least 15-fold higher than manufacturer data collated on the Exeter Universal femoral stem.^8^ Specifically, concerning revision arthroplasty involving the Exeter V40 35.5/125 mm, our report of a stem fracture rate of 8.00% should raise concern.

In 2014, Stryker introduced three new Exeter V40 shorter 125 mm stems to the global market: the 37.5/1/125, 44/1/125 and 50/1/125, all with increased offset to the previous 35.5/125 prosthesis and increased cross-sectional area to the 44/00/125.^36^ Hypothetically, this new range may provide a reduced risk of stem fracture in the 125 mm stem length range, although to date there is no evidence within the literature and we present little data regarding these new stems (Table VIII).

This study has several limitations. First, metallurgical data analysis for this cohort of femoral prosthetic fracture was not obtained. Second, while this study contributes data to better assess incidence, the data represents a minimum institutional incidence. All fractured stems were implanted by surgeons at our centre, however not all implanted stems were followed-up. Finally, this study was limited by the heterogenous nature of the cohort included; indication for primary surgery ranging from inflammatory or osteoarthritis, hip dysplasia, and fractures, as well as inclusion of primary and revision stem fractures.

This case series in conjunction with the systematic review provides evidence stem morphology plays a role in femoral implant fracture. This complication remains rare, although we report a significantly higher incidence at up to 17 years follow-up than in the literature, particularly concerning revision arthroplasty involving the Exeter V40 35.5/125 mm stem. As femoral geometries remain the same, increasing BMIs in THA patients should raise concern. Short 125 mm length Exeter V40 stems undoubtedly have a role in restoring anatomy and biomechanics in smaller femoral geometries, although the surgeon has to appreciate the higher-risk of stem fracture and the associated predisposing factors which necessitate particular attention to surgical technique and planning.

**Take home message**

- Stem morphology plays a role in Exeter V40 stem fracture.

- Risk is significantly higher in the Exeter V40 shorter 125 mm length stems (35.5/125 & 44/00/125).

- We report an 8% stem fracture rate involving revision arthroplasty using the Exeter V40 35.5/125 mm.

- Although this complication remains rare, we report a significantly higher incidence of stem fracture at up to 17-year follow-up than in the literature.
